# Withdrawal: Urinary exosomal tRNA‐derived small RNA signatures as a predictive biomarker in lung cancer

**DOI:** 10.1002/2211-5463.13716

**Published:** 2024-03-19

## Abstract

**Withdrawal:** Urinary exosomal tRNA‐derived small RNA signatures as a predictive biomarker in lung cancer, published in FEBS OPEN BIO, 10.1002/2211‐5463.13716. The above article, published online on 9th October 2023 in Wiley Online Library (wileyonlinelibrary.com), has been withdrawn by agreement between the journal Editor in Chief, Miguel De la Rosa, and Wiley Periodicals LLC. The withdrawal has been agreed following no response from the author to requests to sign the Journal's publishing license/to requests to approve their article proof for publication. https://doi.org/10.1002/2211-5463.13716

